# 

**Published:** 1898-06

**Authors:** 


					CONTENTS.
page!
s?lation after Diphtheria.
By
Bertram M. H. Rogers, B.A.,
M-D.t B.ch. Oxon
101
a J?cclpltal Presentations.
By J.
. Swayne. M.D. Lond....
108
Suggestion for the Treatment of
Jjraves's Disease.
By W. Macphun
temple, M.B., C.M. Glasg.
114
fh6 ?,esenrt>ling in Many Respects
ine Condition known as Mucous
Colitis.
By E. G.Trevithick, M.A.,
^?O., B.C. Cantab., M.R.C.S. Eng.
120
?1:, An Exalted Surgeon and
fnysician.
By Frank Bushnell,
B.S. Lond., M.R.C.S. Eng....
123
Progress of the Medical Sciences:
Medicine.
G. Parker, M.D. ...
126
Surgery.
Charles A. Morton ;
James Swain, M.D.
132
Obstetrics.
Walter C. Swayne,
M.D.
139
Reviews of Books
145
Notes on Preparations for the sick
176
Meetings of Societies
178
The Library of the Bristol Medico-
Chirurgical Society ... ..  I86
Local Medical Notes  193
Scraps
195

				

